# Use of image‐guided robotic‐assisted drilling for transcondylar screw placement in the canine humerus

**DOI:** 10.1111/vsu.70010

**Published:** 2025-09-05

**Authors:** Joshua T. Kershaw, Daniel E. Larby, Fulvio Forni, Matthew J. Allen

**Affiliations:** ^1^ Department of Veterinary Medicine University of Cambridge Cambridge UK; ^2^ Department of Engineering University of Cambridge Cambridge UK

## Abstract

**Objective:**

To determine if a novel robotic system has comparable positional and angular accuracy to that achievable with patient‐specific guides (PSG) when used for transcondylar screw (TCS) placement in the canine humerus.

**Study design:**

Experimental laboratory study.

**Sample population:**

A total of 32 synthetic humeral models (16 per group).

**Methods:**

Bone models were three‐dimensional (3D)‐printed and drilled with the aid of a custom PSG or with the assistance of an image‐guided surgical robot. A 2.5‐mm hole was drilled medial to lateral and the entry point, exit point and angular trajectory of the drill hole were measured on postoperative computed tomography (CT) scans. Absolute differences between planned and actual positions and trajectories were compared between PSG and Robot groups.

**Results:**

None of the drill holes in this study violated the articular surface of the humerus. Entry point positioning was significantly more accurate in the PSG group, but drill hole trajectories (angulation) were more accurate in the Robot group. Exit point positioning was similar in the two groups.

**Conclusion:**

Robotic assistance enables safe placement of drill holes for TCS. PSG enable more accurate drill entry, but robotic assistance allows for more accurate overall drill hole trajectory.

**Clinical significance:**

Robotic assistance allows for accurate and safe drilling of screw holes for TCS placement in the humerus. The robotic procedure allows for a more limited surgical exposure, but the technical feasibility and outcomes associated with this approach should now be evaluated in cadavers before moving to clinical evaluation in live patients.

## 1 INTRODUCTION

Humeral intracondylar fissure (HIF) was first described in 1989^1^ as an intermittent forelimb lameness seen commonly in Cocker Spaniels and French Bulldogs.^2^, ^3^ Surgical treatment of HIF has traditionally involved placement of a positional or lag screw^4^ from either a medial or lateral direction. Previous studies have suggested a lower risk of postoperative complications such as seroma formation and surgical site infection (SSI) with medial‐to‐lateral drilling,^2^, ^5^, ^6^, ^7^ although this is associated with an increased risk of intra‐articular penetration.^2^, ^5^, ^6^

Given the risks of this procedure, efforts have been focused on increasing both the accuracy and the consistency (precision, or repeatability) of transcondylar screw (TCS) placement, and in minimizing surgical time to reduce the risk of SSI. Previous studies have explored the utility of aiming devices^8^, ^9^ and intraoperative fluoroscopy,^7^, ^8^, ^10^ but more recent work has demonstrated the technical superiority of patient‐specific guides (PSG) in the accuracy of TCS placement.^11^ While promising in terms of technical performance, the use of PSG may require a more extensive surgical exposure than a simple aiming guide to ensure accurate apposition of the PSG against the cortical surface. The ideal solution would be to (1) replicate the accuracy of PSG without the need for additional surgical exposure, (2) minimize operative time, and (3) minimize the risk of postoperative complications. To this end, our group has developed a novel robotic solution to support the surgeon in accurate TCS placement through a more limited surgical incision than is typically used for PSG.

The first robot designed for use in orthopedics, ROBODOC, was developed in the early 1990s,^12^ and a number of additional systems have subsequently made it to clinical use in total joint replacement and spinal surgery in human patients.^13^, ^14^, ^15^, ^16^, ^17^ While the development of these systems typically involves establishing safety and feasibility in animal models,^12^, ^18^, ^19^, ^20^, ^21^, ^22^ the high cost of these systems remains prohibitive for veterinary practices. For this project, we elected to focus on the development of a robotic system that is affordable for veterinary surgical specialists. To that end, we paired the robot effector with an existing, commercially available optical tracking system, the performance of which has already been well documented in laboratory studies^23^, ^24^, ^25^ as well as in clinical procedures in human patients.^26^ Other groups are currently working on robotic‐ assisted solutions for veterinary applications, including reduction and fixation of sacroiliac luxation,^27^ but none of these system is currently commercially available to veterinarians.

The goal of the current study was to define the technical performance of image‐guided robotic‐assisted drilling for TCS and to make direct comparisons with the results achievable with PSG. The effectiveness of PSG has already been established, both for TCS placement and other orthopedic procedures.^28^, ^29^, ^30^, ^31^, ^32^ We hypothesized that use of robotic assistance would allow the surgeon to drill holes as safely and as accurately as is achievable with a PSG.

## 2 MATERIALS AND METHODS

### 2.1 Preoperative planning

**FIGURE 1:**
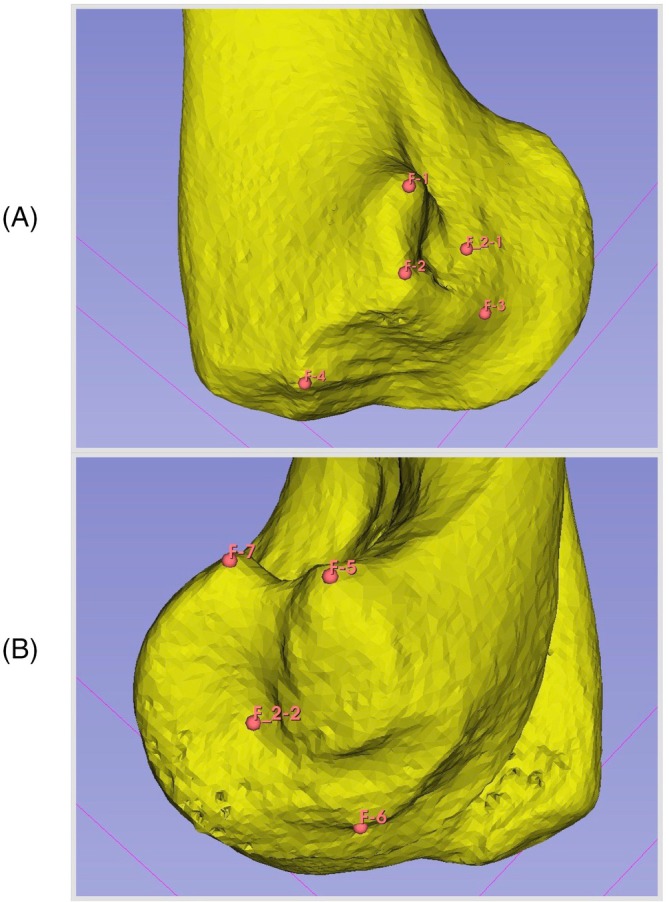
The locations of digitized registration points placed in Slicer on the medial epicondyle (A, F1‐4) and lateral epicondyle (B, F5‐7). Planned entry (F_2‐1) and exit (F_2‐2) are also shown on the medial and lateral epicondyle, respectively.

With the informed consent of the owner, a computed tomography (CT) scan was acquired for the left humerus of a skeletally mature, medium‐sized dog with no evidence of elbow joint disease. A three‐dimensional (3D) model of the humerus was built using open‐source software (Slicer 3D version 5.6.2 [2024]) and surgical planning was then performed in the same software. The planning process defined the optimal entry and exit points for drilling, as well as seven clearly defined anatomical landmarks for use in registration of the surgical robot. Entry and exit points were based on published recommendations.^28^ The landmarks, all located adjacent to the medial or lateral epicondyles of the humerus, were selected on the basis that they were easily palpable through the intact skin over the elbow joint (Figure 1). The x, y and z coordinates of the seven landmarks and the planned entry and exit points were exported as .*json* files for use by the robotic system. The same points were also exported as 3D coordinate data for use in 3D design software (see below).

### 2.2 Production of PSGs

**FIGURE 2:**
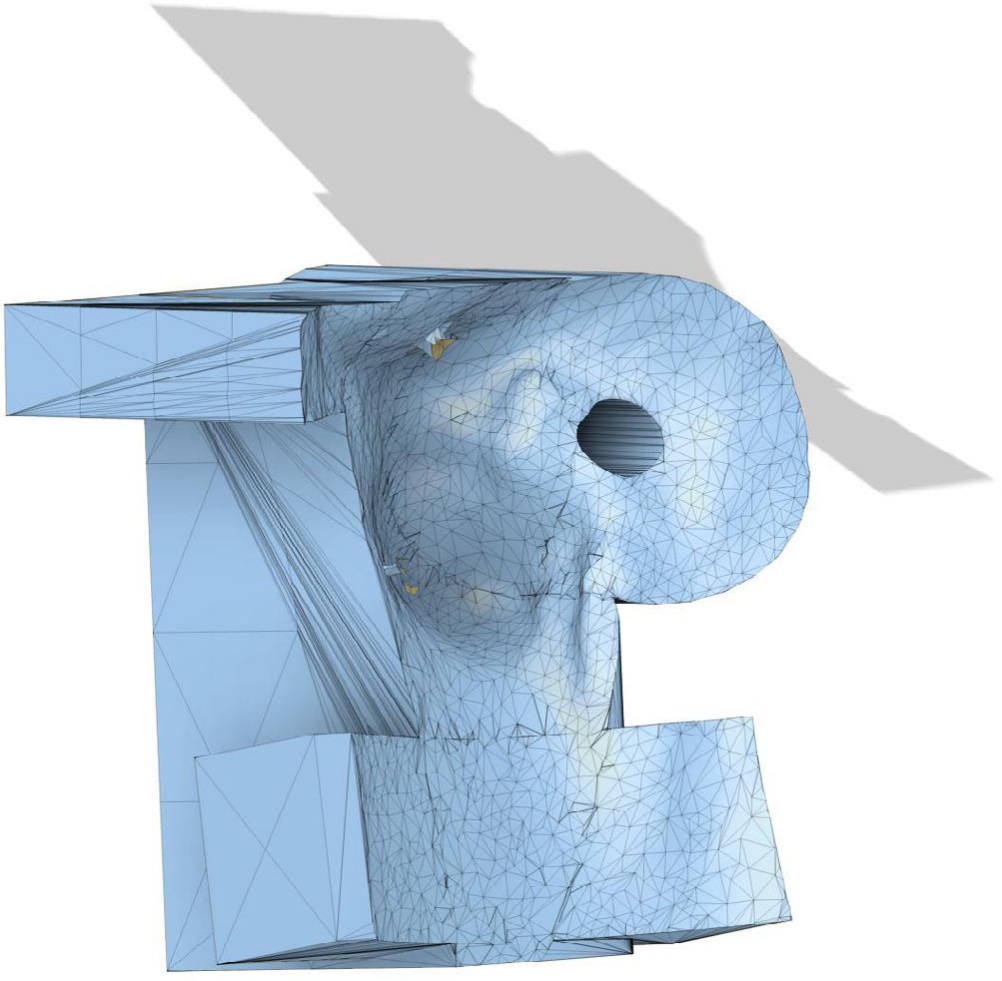
Design of the printed patient‐specific guide (PSG) used in the procedures. The PSG was manufactured using a stereolithography (SLA) printer and autoclavable resin.

The entry and exit points were imported into open‐source software (Blender version 4.2.3; Blender Foundation., Amsterdam, Netherlands) and converted into a cylinder (Ø 2.5 mm) that represented the planned pilot drill hole. The drill hole was resized to 5 mm to ensure that it did not penetrate the articular surface at any point. The PSG was then constructed by designing a drill sleeve around the original 2.5‐mm cylinder, then fusing this with a baseplate that closely matched the external contours of the medial epicondyle.^11^ Final editing of the guide to remove any excess material was performed in Autodesk Fusion (Autodesk Fusion version 2.0.16490; Autodesk Inc., San Francisco, California). The guides were printed on a desktop stereolithography (SLA) printer (form 2 SLA 3D printer; Formlabs Inc., Somerville, Massachusetts) using a proprietary resin that is tissue compatible, autoclavable and suitable for clinical use in the form of patient‐specific surgical guides. Layer height for the PSGs was 0.05 mm. The design of the printed PSG is shown in Figure 2.

### 2.3 Creation of bone models

**FIGURE 3:**
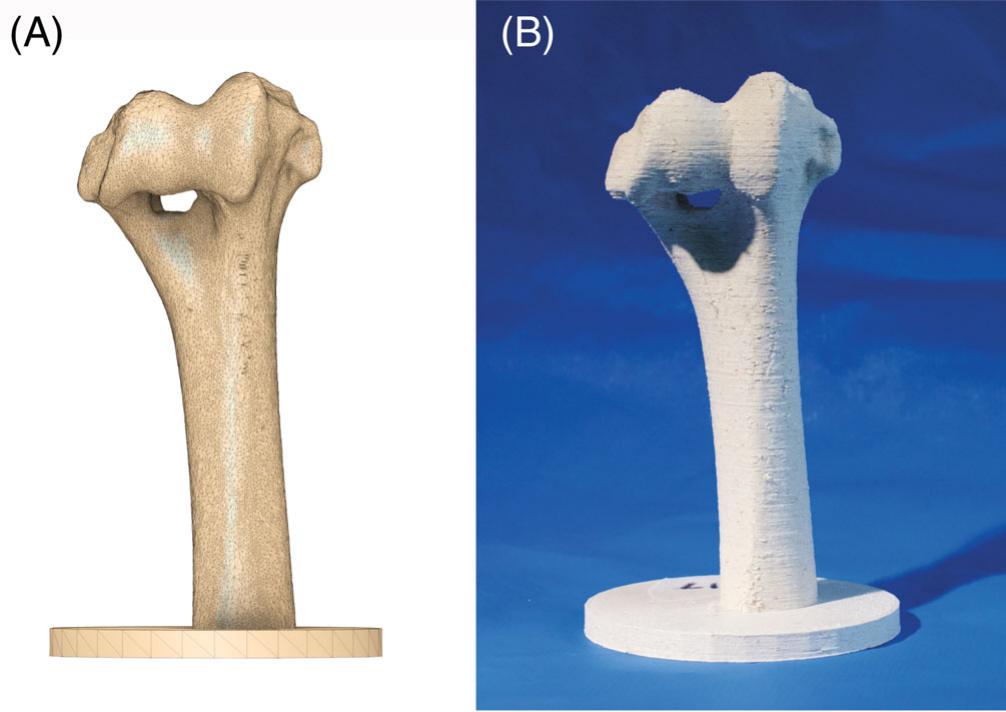
(A) Three‐dimensional (3D) rendering of the bone model design produced in Autodesk fusion. (B) Printed model made from the wood‐PLA mix material.

The bone models were also produced using Blender and Autodesk Fusion. The bone model was cropped to include just the distal half of the humerus, and a circular plate was added to its base to act as a support (Figure 3A). The bone models were then printed using a desktop fused deposition modeling (FDM) 3D printer (Prusa Mark 3S printer; Prusa Research a.s., Prague, Czech Republic) and a wood‐PLA composite resin filament (RS PRO Dark Wood 3D Printer Filament; RS Components, UK). This material was chosen to avoid any melting or warping of the models during drilling, as this could have affected the accuracy of the procedure and/or analysis. Layer height was set to 0.2 mm and a 0.4 mm nozzle was used. The final printed model is shown in Figure 3B. An a‐priori power analysis determined that a sample size of 16 bone models per group would be needed to determine a 1‐mm difference between the groups (α = 80%, *p* < .05). The threshold of 1 mm was based on previous work on PSG in canine cadavers.^11^

### 2.4 Robot control mechanism

**FIGURE 4:**
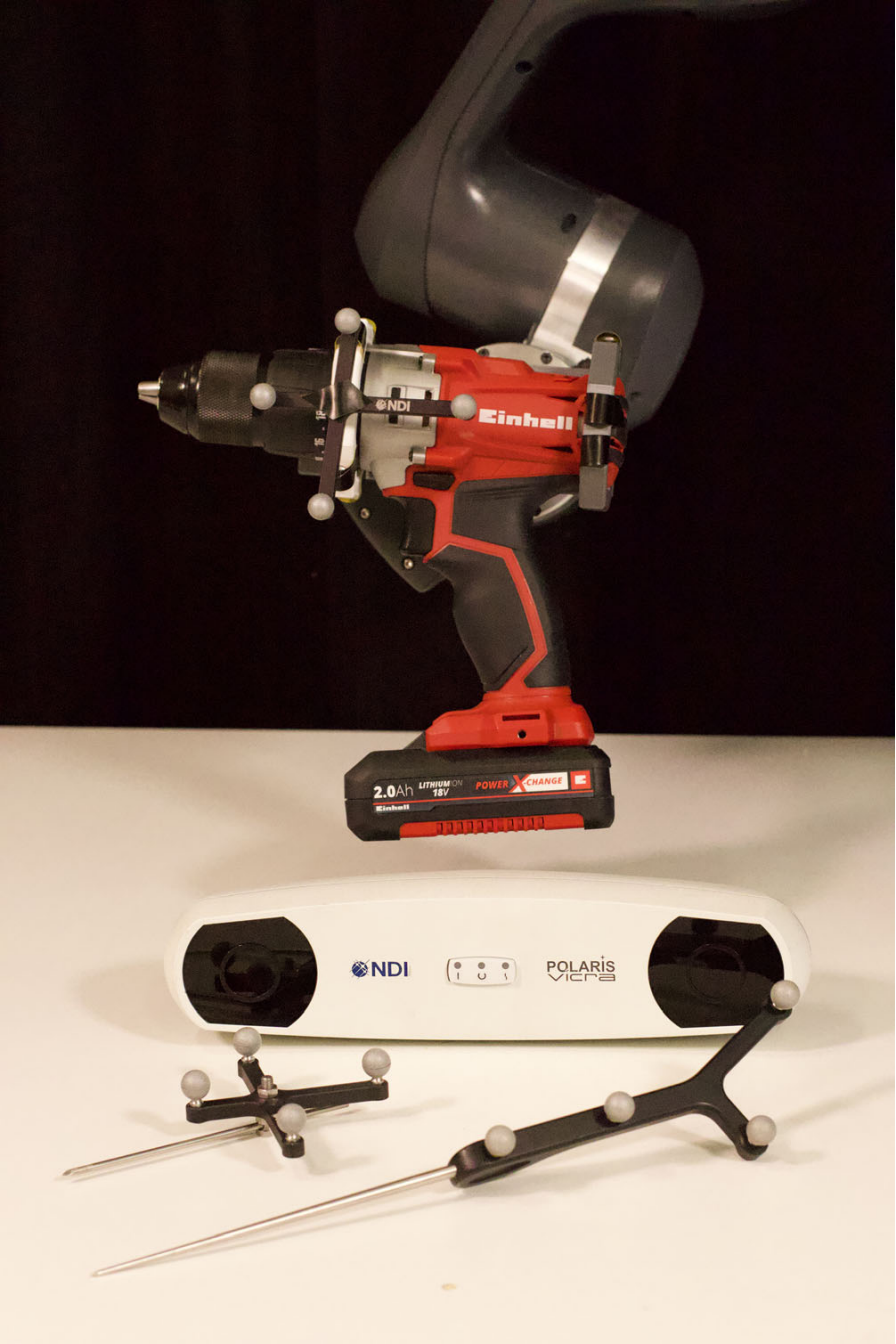
Set up of the optical tracking system. The camera and associated markers located on the; scaffold holding the drill, a 3‐mm Ellis pin is placed in the humeral diaphysis and a pointer used to mark registration points.

The robot control mechanism used in this study has been reported previously^33^ and will not be described in detail here. In brief, the robotic system consists of a robotic manipulator (Franka Research 3 manipulator; Franka Robotics GmbH, Munich, Germany), a 3D optical tracking system (Polaris Vicra; Northern Digital Inc., Waterloo, Ontario, Canada), a digitizing stylus, and retroreflective optical assembly that is rigidly fixed to the bone that is being tracked (Figure 4).

**FIGURE 5:**
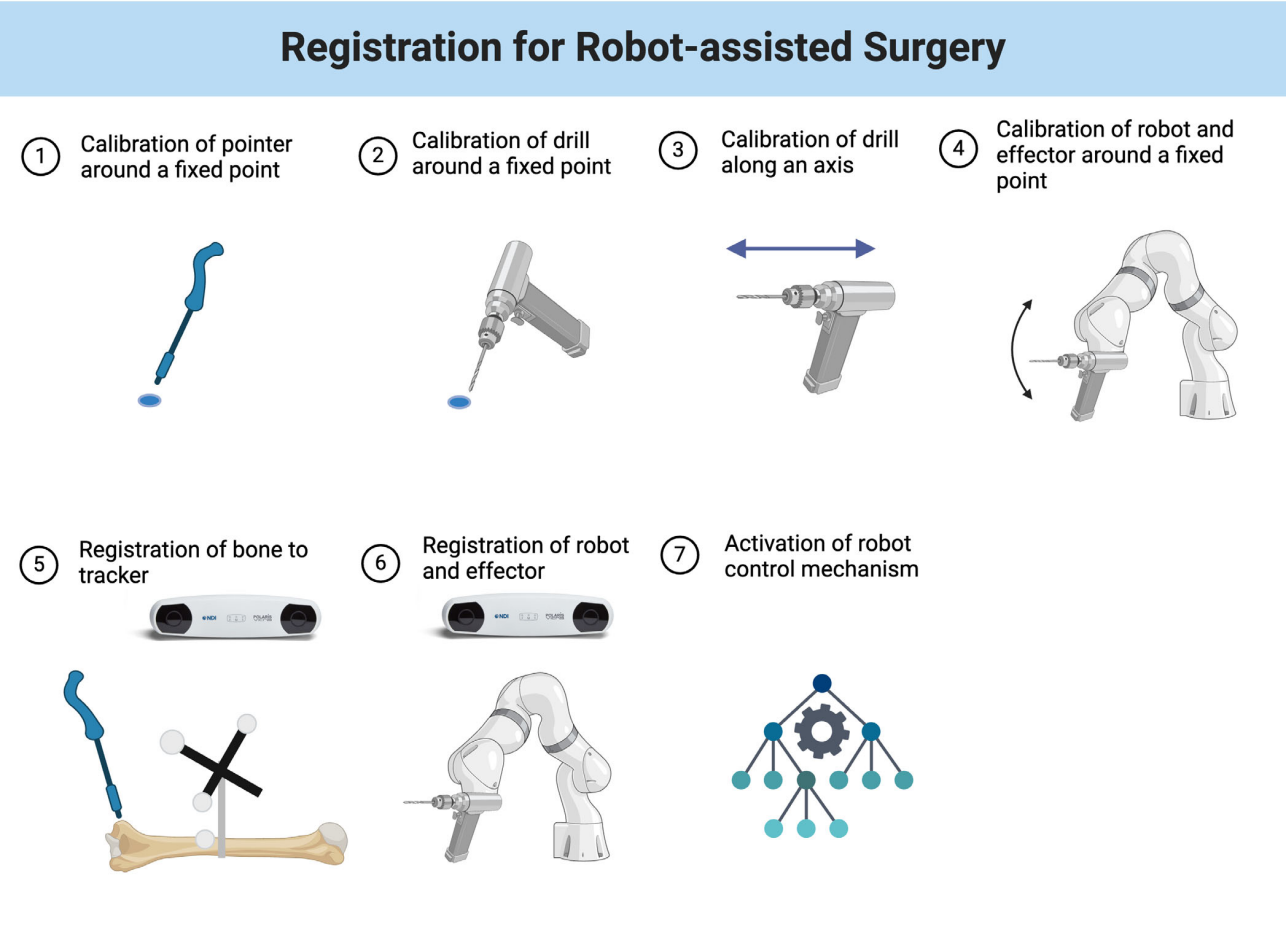
Workflow for robotic‐assisted procedures. First a referencing pointer is calibrated around a fixed point, measuring for 30 s. A drill is then calibrated in the same way. In addition, the axis of the drill is calibrated by moving it along a fixed axis for 30 s The drill is then attached to the robotic arm and steps 2 and 3 are repeated. The bone is then registered using the points shown in Figure 1 and the pointer, the final stage is the registration of the robot in the frame of view of the optical tracking camera. This allows for all necessary transforms to be appropriately calculated.

The steps for robot set up are outlined in Figure 5.

Steps 1–4 (Calibration) were conducted once so remained constant, these are independent of camera position, while steps 5–7 were repeated for every model. A maximum threshold of a 1.5 mm root mean square (rms) error was set in the registration of the bone to be deemed acceptable, and the registration was checked using the pointer and a live 3D plot of the bone.

### 2.5 Surgical procedure

**FIGURE 6:**
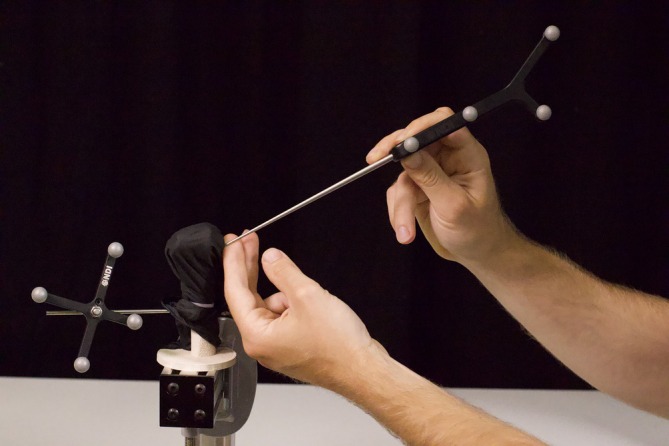
Bone registration of predetermined registration points (outlined in Figure 1), five measurements were taken at each of the seven landmarks, and the whole process was then repeated three times to produce the fit (105 points). A point matching method and a rigid best fit transform were used to model the location of the bone within the volume of the optical tracking system. The anatomical landmarks are obscured using a latex sleeve so the surgeon is unable to visualize them.

**FIGURE 7:**
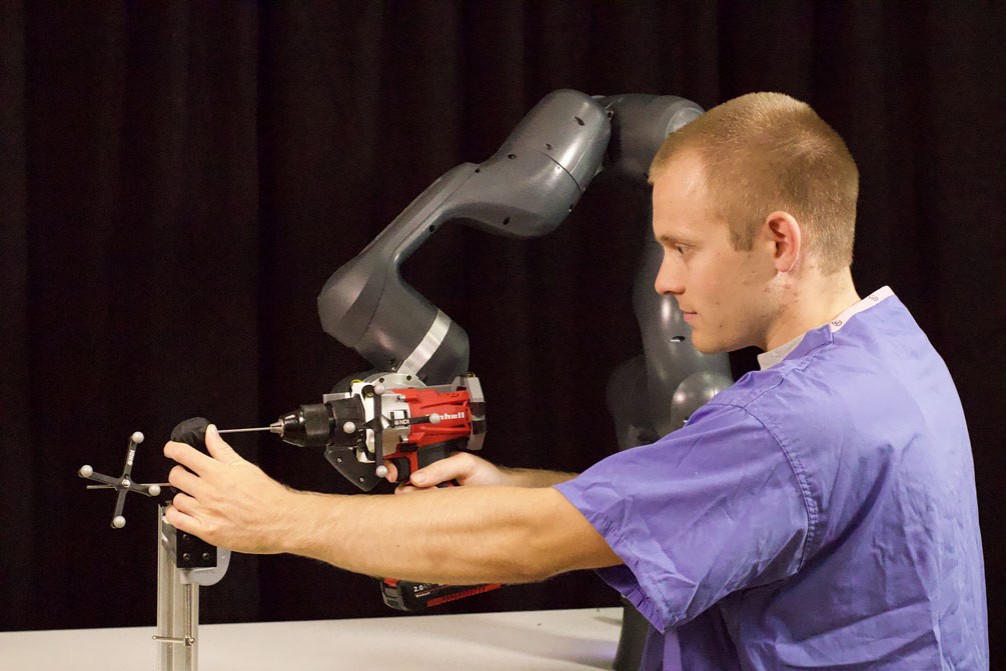
The surgeon uses the robotic system to drill across the humeral condyle. The tip of the drill is supported at the point of entry to prevent excessive cranial slip of the drill on the medial epicondyle. The other hand stabilizes the bone to prevent excessive movement and to limit the requirement for the robot to continuously update the trajectory during drilling.

In a clinical setting, the anatomy and landmarks of the distal humerus are covered with skin and soft tissues. A latex covering was therefore applied over the bone model to mask the contours and landmarks over the epicondyles (Figure 6). The model was clamped to simulate the dog in dorsal recumbency to facilitate registration on both condyles. For robot‐assisted surgeries the procedure outlined above was followed and the control mechanism activated. The surgeon then checked the alignment of the drill, and an assistant confirmed live outputs from the mechanism (tracker visible on bone, tracker visible on drill, mechanism integrator control not saturated). Drilling was then initiated by gently guiding the drill along the planned trajectory. The bone model was supported with the surgeon's non‐drilling hand and an initial entry hole was established. The hole was then completed by drilling across the condyle. The behavior of the robot and any complications were noted. The robot‐surgeon interface is shown in Figure 7.

**FIGURE 8:**
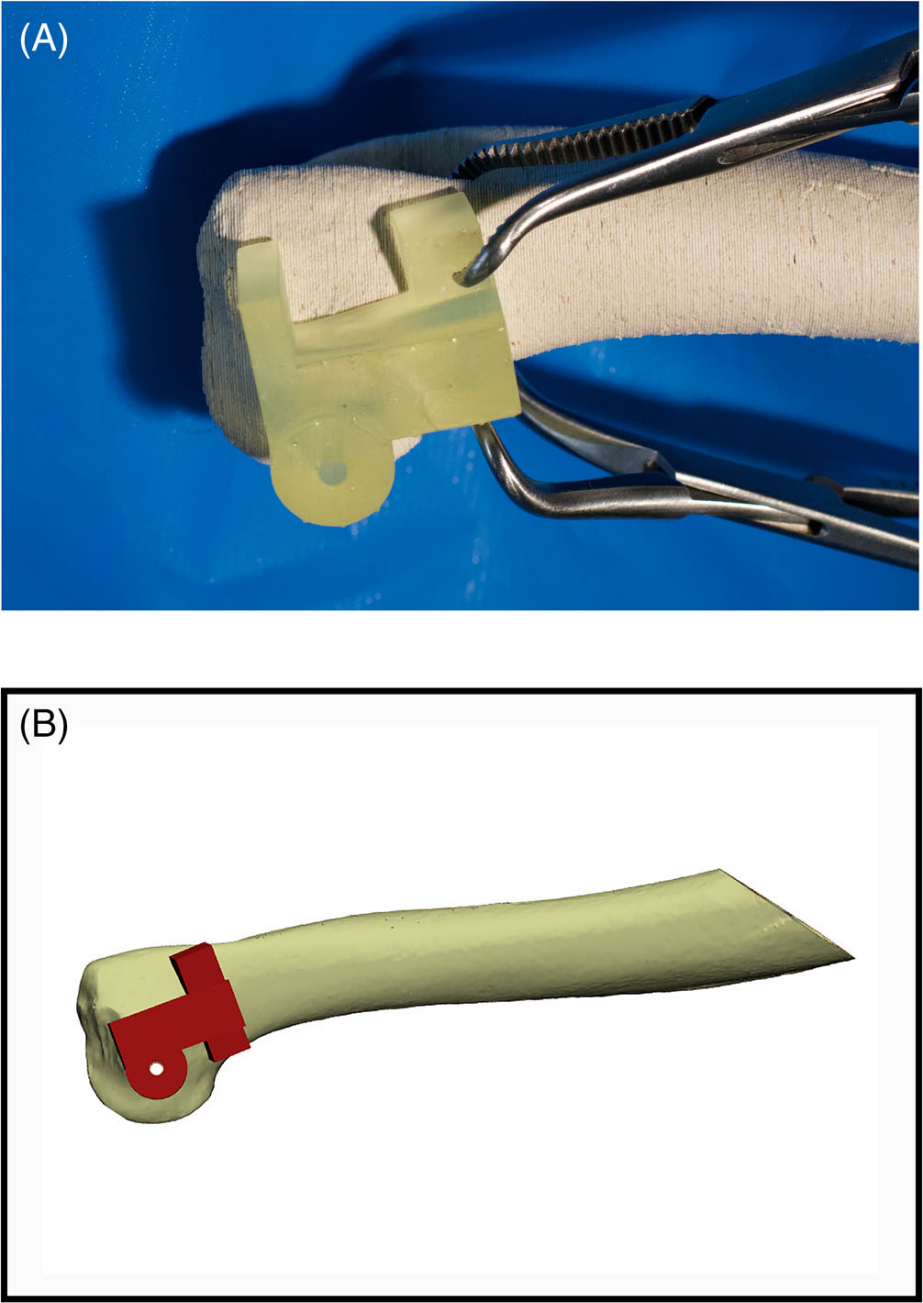
(A) Set up for patient‐specific guide (PSG) surgeries, with the humeral model in simulated left lateral recumbency and clamped using a worktop clamp. Pointed reduction forceps are placed onto the “wings” of the printed guide and the lateral aspect of the medial condyle of the bone to rigidly hold the PSG in place during drilling. (B) CAD image of the PSG seated on the bone surface.

For the PSG group, the bone model was clamped in left lateral recumbency. The PSG was secured to the bone surface with pointed reduction forceps (Figure 8) that prevented relative movement of the PSG over the model. Care was taken to ensure that placement of the reduction forceps did not result in lifting of the PSG off the bone surface. Once accurate seating and alignment of the PSG had been confirmed, the hole was drilled across the condyle as above, noting any complications.

A brad point drill bit (StickTite; IMEX; Longview, Texas) was used in this study because pilot work had identified the potential for the drill bit to slip on the irregular surface of the medial humeral epicondyle. A similar effect had previously been noted with the use of a manual aiming device.^11^

### 2.6 Imaging

Postoperative CT imaging was performed on a 16‐slice clinical CT scanner (Toshiba Aquilion; Toshiba Medical Systems, London, UK) operating at 100 kV and 80 mA with a slice thickness of 0.5 mm. For scanning, the humeral models were placed in simulated sternal recumbency. The trajectory of the drill hole across the condyle was marked by placing an aluminium rod (Ø 2.5 mm) along the length of the drill hole. The resulting helical axial CT images were reconstructed using a proprietary bone algorithm.

### 2.7 Postoperative assessment

Postoperative scans of the drilled humeri were imported as DICOM images into medical modeling software (MIMICS version 26.0; Materialize BV, Belgium) and 3D models were created using the global threshold function. Separate models were created using discrete thresholding profiles for (1) bone and (2) the aluminium rod (representing the trajectory of the drill hole). The two models were then exported in STL format into CAD‐based design/analysis software (3‐Matic version 16.0; Materialize BV).

The preoperative planned trajectory and associated 3D in‐silico model were also imported into 3‐Matic and aligned so that the world coordinate system represented the anatomical coordinate system of the humerus. The “n‐points registration” feature was then used to align the bones, and the “global registration” feature was used to ensure optimal alignment. The cylinders were marked, and for the drilled trajectory a best fit cylinder was produced from which an inertial axis could be generated. The planned cylinder had its central axis defined as a line by fitting to its axes of inertia.

The accuracy of drill entry and exit points was determined by measuring the distance (in millimeters [mm]) between points at the centers of the drill holes in the medial and lateral cortices for both the planned and the achieved drill holes.^11^ The accuracy of the drill hole trajectory was calculated as the difference (in degrees [°]) between the angulation of the planned and achieved drill holes. This was performed in 3‐Matic using the “Measure” feature. Angular errors were measured in terms of both the local coordinate system (i.e., as differences in x, y and z orientation within the 3D space) and an anatomical coordinate system based on landmarks on the humerus. In this anatomical coordinate system, the x‐axis represents the medial‐to‐lateral axis of the humerus, the y‐axis represents the longitudinal axis, and the z‐axis represents the cranial‐to‐caudal axis.

To assess safety, defined here as avoidance of inadvertent joint penetration, the diameter of the cylinder representing the drill hole was expanded to a diameter of 5 mm to represent a 5‐mm TCS placed across the condyle. The images were then evaluated to see if the 5‐mm cylinder violated the articular surface, and the outcome (Yes/No) was recorded for both treatment groups.

### 2.8 Data handling and statistical analysis

Descriptive data were generated for all specimens and reported as the mean and standard deviation. For continuous data relating to accuracy, comparisons between PSG and Robot specimens were evaluated using a paired Student's *t*‐test. Precision was evaluated by use of a precision analysis. A parametric second‐order analysis was conducted on the angular distribution of entry, exit and angular measurements to better define differences between the spatial/anatomic distribution of drill holes in the two groups. The incidence of joint penetration in the two groups was compared using a Fisher's exact test. Significance was set at *p* < .05 for all analyses.

## 3 RESULTS

All models drilled in this study were deemed to have TCS placement that would have been “safe”, with no instances of intra‐articular penetration noted.

**FIGURE 9:**
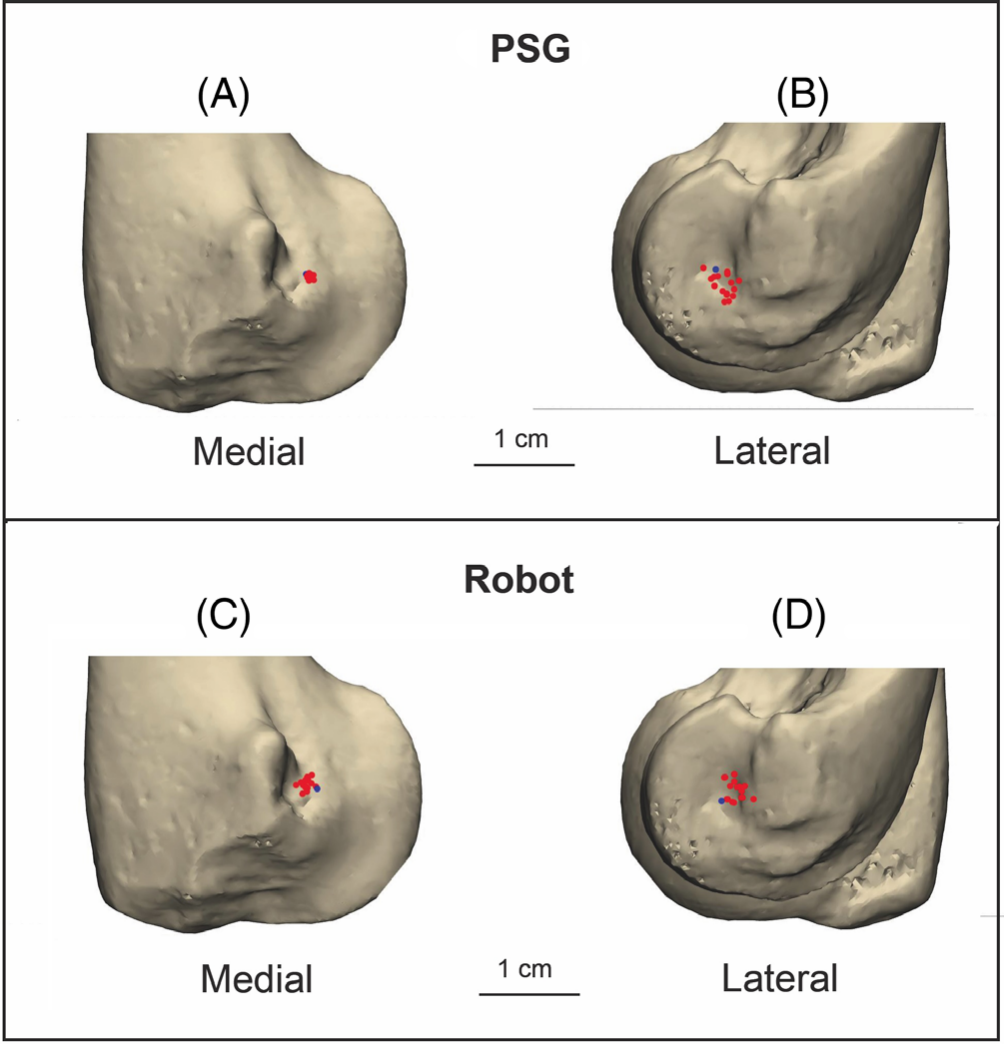
Entry points (A) and exit points (B) achieved in the patient‐specific guide (PSG) group, compared with entry points (C) and exit points (D) in the Robot group. In each plot, the red dots indicate the locations achieved in surgery, and the blue dots represent the positions anticipated from the preoperative plan.

**FIGURE 10:**
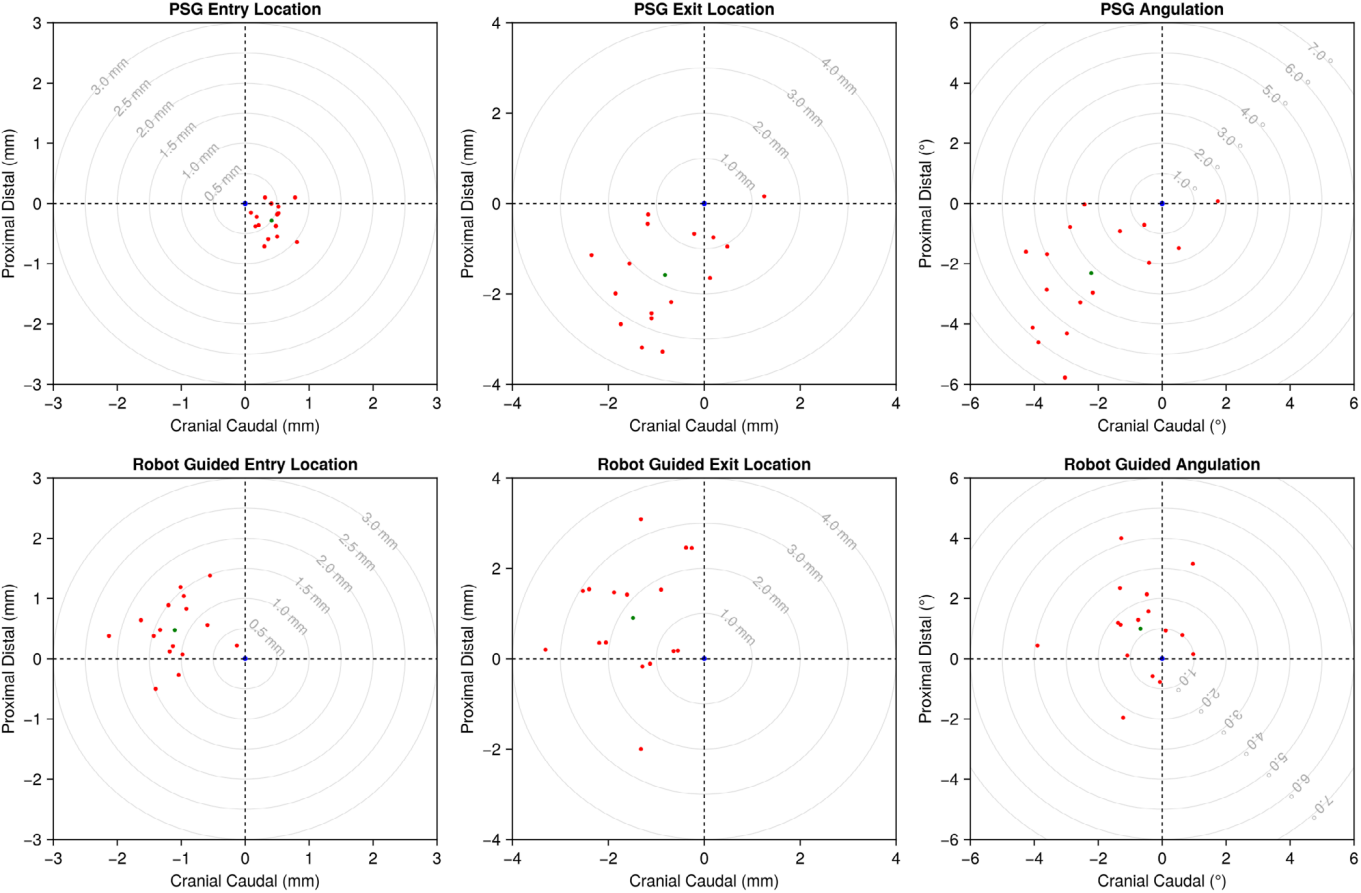
Polar plots illustrating actual (red) and planned (blue) entry points, exit points and angular trajectories. For each surgical method, patient‐specific guide (PSG) and Robot, mean values are shown in green. In this figure, cranial and proximal are positive, caudal and distal are negative.

Absolute errors (mean ± SD, mm or °) for entry points, exit points and angular trajectory for drill holes prepared using PSG or the Robot are shown in Table 1. Plots of the entry and exit points relative to those planned preoperatively are presented in Figure 9, providing a visual representation of the spatial/anatomical location of recorded errors. The data are then presented in the form of polar plots in Figure 10 to allow for easier interpretation of the relative magnitude of errors between the two groups.

**TABLE 1:** Absolute errors between planned and actual entry and exit points (in millimeters) and angular trajectory (in degrees) in the 3D volume (xyz angle) and in the two anatomic planes (planar angle – CrCd and planar angle ‐ PrDis). Data are presented for PSG and Robot groups, with *p*‐values reflecting comparisons between the two groups.

	PSG					Robotic assistance				
	Entry	Exit	xyz angle	Planar angle CrCd	Planar angle PrDis	Entry	Exit	xyz angle	Planar angle CrCd	Planar angle PrDis
Mean	0.554	2.06	3.57	2.50	2.32	1.31	2.12	1.90	1.01	1.41
SD	0.220	0.946	1.77	1.28	1.73	0.427	0.829	1.11	0.888	1.08
*p*‐value	NA	NA	NA	NA	NA	**0.0003**	0.890	**0.008**	**0.00131**	0.100

*Note:* Bold indicates statistical significance (*p*‐value < 0.05).

Abbreviations: 3D, three‐dimensional; PSG, patient‐specific guide.

Entry holes drilled with direct support from the PSG were significantly more accurate than those created with the Robot (*p* = .00003). For the PSG group the mean ± SD entry error was 0.41 ± 0.20 mm (i.e., cranial) and − 0.28 ± 0.26 mm (i.e., distal) relative to the planned positions. For the Robot group the mean ± SD directional entry error was −1.10 ± 0.46 mm (i.e., caudal) and 0.48 ± 0.51 mm (i.e., proximal).

There was no statistically significant difference in exit error between the two groups (*p* = .890). For the PSG group the mean ± SD exit error was −0.82 ± 0.96 mm (i.e., caudal) and −1.58 ± 1.07 mm (i.e., distal). For the Robot group, mean ± SD directional exit error was −1.48 ± 0.86 mm (i.e., caudal) and 0.90 ± 1.27 mm (i.e., proximal).

The Robot group showed a statistically significant improvement in the accuracy of drill hole angular trajectory compared to that achieved with PSG (*p* = .008). The mean ± SD errors in angular trajectory were −2.22 ± 1.75° (i.e., caudal) and − 2.31 ± 1.75° (i.e., distal) for the PSG group, compared with −0.68° ± 1.18° (i.e., caudal) and 1.00 ± 1.49° (i.e., proximal) for the Robot group.

Precision or repeatability (i.e., variance from the mean) was not significantly different between the two groups for the exit points (*p* = .812) or for drill hole angular trajectory (*p* = .972). However, PSG did yield a statistically significant improvement in entry point precision as compared with the Robot (*p* < .001).

From a spatial/anatomical perspective (Figure 10), there was a significant difference in the distribution of entry points (*p* < .001), with holes in the PSG group being more caudo‐distal and those in the Robot group more cranio‐proximal. Exit holes in the PSG group were also clustered more caudo‐distally as compared with a more caudo‐proximal distribution in the Robot group (*p* < .001). These differences in entry/exit point distribution were matched with significant differences in angular trajectory.

## 4 DISCUSSION

This study has demonstrated that a custom robotic system, developed specifically for veterinary use, supports safe and accurate drilling in bone. Although there have been previous reports on the use of surgical robots in animal models, these systems were intended for clinical use in human rather than veterinary patients.

The results achieved with PSG in this study replicated previously published data,^11^ confirming the reproducibility of PSG in a laboratory setting. Although the technical superiority of PSG over non‐patient specific drilling techniques has been confirmed,^11^ the potential drawbacks of PSG, including the need for increased surgical exposure and the risk of poor guide seating, cannot be ignored. In the current study the robot‐assisted procedure, performed through a more limited surgical exposure, was found to be safe, accurate and repeatable. While the study did not explicitly focus on the benefits of a less invasive approach offered by image‐guided navigation, our finding that the robotic approach has similar accuracy to a more invasive PSG approach suggests that the robotic system could offer clinical advantages to currently used PSG.

There was no significant difference in drill hole exit position between the two groups, but entry positions for the PSG were significantly more accurate than those drilled with robotic assistance. Previous work^11^ associated a better entry position with a lower risk of articular penetration, however in this study there was no difference in the “risk” of intraarticular joint penetration, and all of the drill holes were classified as “safe”. This may be due to the overall improvement in the trajectory of the drill hole compared to planned by use of the Robot mechanism (Table 1). The improved accuracy of TCS orientation may be especially advantageous in the context of humeral condylar fracture repair, where the risk of screw failure is reduced by positioning the TCS perpendicular to the fissure line.^34^, ^35^ Whilst we did see similar deviations in drill exit position relative to the planned position, the trajectory of robot drilled specimens subjectively appeared closer to that defined in the planning process. This represents the robot's ability to leverage real‐time motion capture to update the trajectory of the drill hole, even if the initial entry position is displaced slightly. Specimens in the Robot group did have a lower variance (*p* = .0013) in cranial‐caudal angulation of the screw but no statistical difference in proximal‐distal angulation.

Drill hole entry positions were less accurate in the Robot group than in the PSG group. It has previously been noted that there is a tendency for the drill bit to slip when drilling on the face of the medial humeral epicondyle.^11^ Although care was taken to avoid slippage or bending of the relatively long 2.5‐mm drill bit used for these surgeries, some degree of inaccuracy is inevitable. Even with robotic assistance there can be a tendency for cranial drift if the drill bit is not stabilized against the bone before drilling starts. Errors in drill hole entry point and angular trajectory could also reflect small errors in bone registration, discussed below. The robot control system is not entirely rigid, as it must react to measured errors, and cannot do so instantly. There are also limits on the total force the robot can apply, as a safety measure. Surgeon training and familiarity must be considered when using this system: they must be aware that slow, controlled motions with minimal tangential forces will give the best results. However, the authors found it was relatively easy to adjust drilling technique to incorporate the robot.

Barnes et al.^35^ has previously defined “safe corridors” for drilling from lateral‐to‐medial and medial‐to‐lateral in the humeral condyle. Current clinical evidence suggests that medial‐to‐lateral drilling offers a lower incidence of postoperative complications^7^ but with a narrower safety envelope. All the drill holes prepared in this study were within this safe envelope.

A previous study on drilling accuracy in the sacral wing reported an average angulation error of between 3 and 4°.^36^ Similar studies have not been carried out on the humeral condyle, but the mean angulation from the robotic system was below 2° (1.90 ± 1.11°) and only one drill hole exceeded 4° (4.2°). The outlier may have been associated with poor bone registration; however, there was no correlation between bone registration and outcome, so it is more likely that the error represented a technical error such as drill slippage. Overall, the data demonstrate that a relatively inexperienced surgeon using this robotic system is capable of matching or exceeding the accuracy of an experienced surgeon performing the surgery with a freehand technique.

The major limitation of this study lies in the use of 3D printed models rather than cadavers. Bone models were used because they make it possible to perform systematic evaluation of technical reproducibility and to make direct comparisons between the performance of the Robot and the PSG. It should be noted that performance of a PSG on a bone model may be superior to that achievable in the clinic, where the positioning and fit of the PSG on bone are critically dependent on the presence or absence of intervening soft tissues. Our results with PSG on the bone model were similar to those reported previously^11^ but we accept that they may be particular to this specific PSG design, which was not manufactured by a commercial vendor. The procedures were not entirely clinically realistic in that landmarks were visually obscured by a barrier material, rather than by skin of variable thickness. In a clinical setting, the selection of landmarks for registration should be based on the reproducibility of locating these landmarks in the live patient – this will be an area of follow‐up study in cadavers.

A systematic error in bone registration was identified in this study. Although anatomical landmarks on the distal humerus were digitized for each specimen in the Robot group, the bone model did not encompass the proximal humerus, and the accuracy of humeral registration could likely have been improved with inclusion of additional, proximal landmarks. Inclusion of proximal landmarks would allow for better matching of the bone surface against the CT, particularly in terms of rotational alignment. In a clinical setting, soft tissue swelling, as might be seen with an elbow fracture, could also reduce the accuracy of registration. These issues could potentially be resolved by the use of a fixed tracker that is secured to the bone and then included in the preoperative CT scan.^12^ This approach would ideally require a CT scanner inside the operating room, something that is expensive and not widely available. Our ongoing work is evaluating the use of fixed bone trackers to see if they improve bone registration and/or the technical accuracy of drilling.

The primary benefit of the use of a robotic system compared to a PSG is the reduced soft tissue exposure required. This could be particularly beneficial in surgeries of the elbow due to the reported incidence of complications such as seroma formation. However, other surgeries could benefit from a more limited approach or when the soft tissues are too extensive for the fitting of surgical guides, for example during total hip replacement. This study proves the effectiveness of using a robot to guide a drill along a defined trajectory; however, more work is required to determine the effectiveness of this approach in veterinary practice and to confirm that the extra steps needed for bone registration do not significantly extend anesthesia time or impact postoperative outcomes.

## 5 CONCLUSIONS

This study has established the safety and technical accuracy of a robotic system for assisted drilling of transcondylar screws in model canine humeri. Results with the robot‐assisted procedure were as good as those achievable with a PSG and could be performed through a less extensive surgical incision. Additional work is now needed to further improve bone registration and drill stability, and to determine the performance of the system in cadavers, before moving into potential clinical trials in patients.

## AUTHOR CONTRIBUTIONS

Kershaw JT, BA, Larby DE, MA, Forni F, PhD and Allen MJ, VetMB, PhD, MRCVS: Were involved in the development of this system and the design of this study. Kershaw JT, BA and Larby DE, MA: Performed all procedures together. Kershaw JT, BA: Planned the procedures and conducted postoperative measurements. All authors contributed to the final write up of this work.

## FUNDING INFORMATION

This work was funded by two intramural grants from the relevant departments within the University of Cambridge.

## CONFLICT OF INTEREST STATEMENT

The authors declare no conflicts of interest related to this report.
